# Supplementary material for: Changes in microRNA Expression in the Cochlear Nucleus and Inferior Colliculus after Acute Noise-Induced Hearing Loss
Source: Int J Mol Sci. 2020 Nov 20;21(22):8792. doi: 10.3390/ijms21228792 (PMC7709026; doi:10.3390/ijms21228792)
Supplement: Supplementary file 1 [file ijms-21-08792-s001.pdf]

Color index			
Log(2) ratio	Fold	Color	RGB
>3.321928095	>10		150.5.1
>2.9068906	>7.5		200.7.2
>2.321928095	>5		250.8.2
>1.5849625	>3		253.52.47
>1	>2		253.102.98
>0.8073549	>1.75		254.152.148
>0.584962501	>1.5		255.202.201
>0.3219281	>1.25		255.230.229
0	1		255.255.255
<-0.3219281	<0.8		232.229.255
<-0.584962501	<0.6666666		205.205.205
<-0.8073549	<0.5714286		152.162.254
<-1	<0.5		88.104.254
<-1.5849625	<0.3333333		39.59.253
<-2.321928095	<0.2		2.25.244
<-2.9068906	<0.1333333		1.14.137
<-3.321928095	<0.1		1.9.83

Figure S1: Color index of fold change for candidate miRNAs.

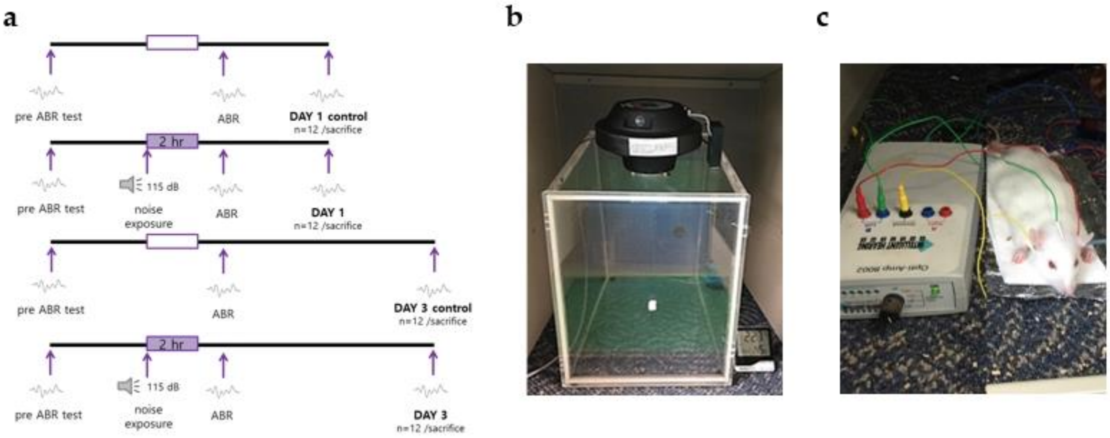

Figure S2: Noise exposure experiment. (a) Noise exposure protocol. Empty boxes indicate control condition without noise. (b) Animals were anesthetized and separated during noise exposure. (c) An anesthetized animal during ABR test.

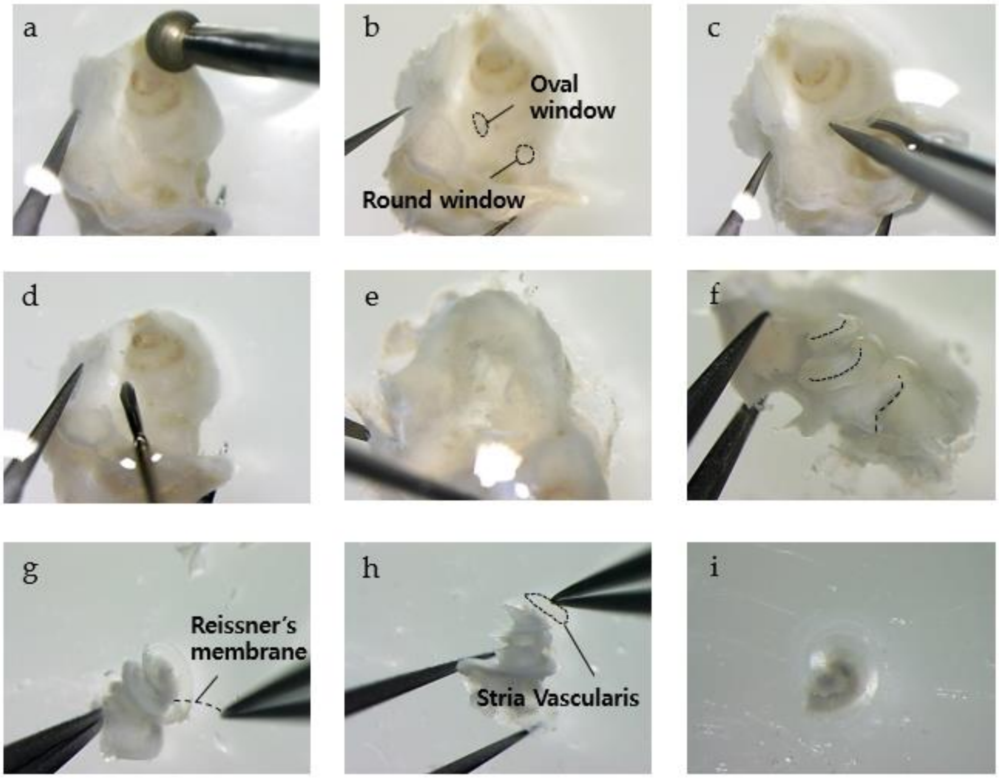

Figure S3: The cochlear whole-mount surface preparation procedure. (a) Drilling the laminar bone that covers the cochlea. (b) and (c) Breaking the bone between the oval and round windows. (d), (e), and (f) Isolating the spiral structure from the organ of Corti. (g) and (h) Removing the Reissner's membrane and stria vascularis. (i) The apical turn section that was removed from the top.

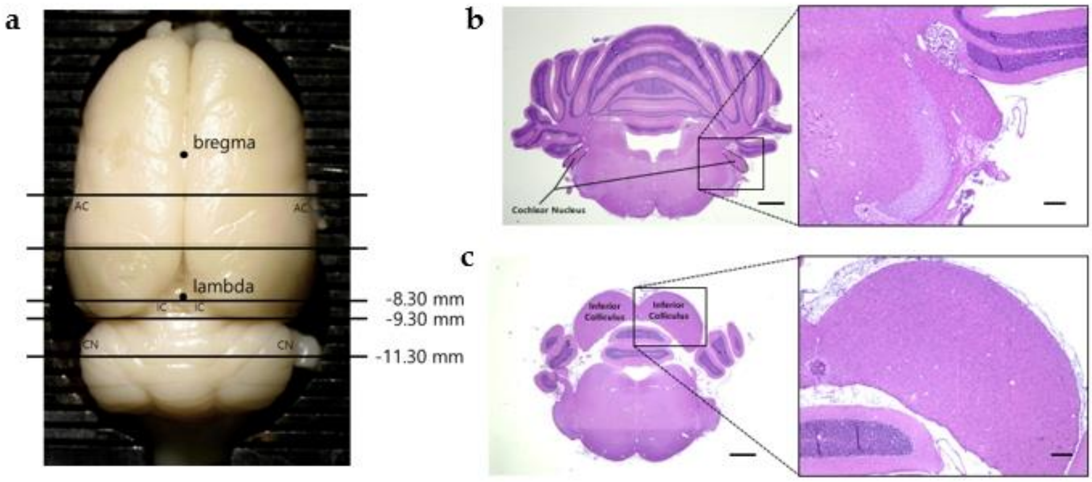

Figure S4: Locations of the CN and IC. (a) Cutting location for the IC and CN on a stainless brain matrix. (b) Location of the IC based on the rat brain atlas. (c) Location of the CN based on the rat brain atlas.
